# The Role of Proteomics in Biomarker Development for Improved Patient Diagnosis and Clinical Decision Making in Prostate Cancer

**DOI:** 10.3390/diagnostics6030027

**Published:** 2016-07-18

**Authors:** Claire L. Tonry, Emma Leacy, Cinzia Raso, Stephen P. Finn, John Armstrong, Stephen R. Pennington

**Affiliations:** 1 UCD Conway Institute of Biomolecular and Biomedical Research, University College Dublin, Dublin 4, Ireland; claire.tonry@ucdconnect.ie (E.L.); cinzia.raso@ucd.ie (C.R.); 2 School of Medicine, Trinity College Dublin, Dublin 2, Ireland; stephen.finn@tcd.ie; 3 St. Luke’s Hospital, Rathgar, Dublin 6, Ireland; john.armstrong@slh.ie

**Keywords:** prostate cancer, proteomics, biomarkers, multiple reaction monitoring, clinical validation

## Abstract

Prostate Cancer (PCa) is the second most commonly diagnosed cancer in men worldwide. Although increased expression of prostate-specific antigen (PSA) is an effective indicator for the recurrence of PCa, its intended use as a screening marker for PCa is of considerable controversy. Recent research efforts in the field of PCa biomarkers have focused on the identification of tissue and fluid-based biomarkers that would be better able to stratify those individuals diagnosed with PCa who (i) might best receive no treatment (active surveillance of the disease); (ii) would benefit from existing treatments; or (iii) those who are likely to succumb to disease recurrence and/or have aggressive disease. The growing demand for better prostate cancer biomarkers has coincided with the development of improved discovery and evaluation technologies for multiplexed measurement of proteins in bio-fluids and tissues. This review aims to (i) provide an overview of these technologies as well as describe some of the candidate PCa protein biomarkers that have been discovered using them; (ii) address some of the general limitations in the clinical evaluation and validation of protein biomarkers; and (iii) make recommendations for strategies that could be adopted to improve the successful development of protein biomarkers to deliver improvements in personalized PCa patient decision making.

## 1. Introduction

Most men will develop prostate cancer (PCa) if they live long enough [1]. Indeed, population-adjusted figures show that PCa is the fifth most common cause of cancer-related death for males worldwide [2,3]. PCa is a disease of the elderly, as the likelihood of developing it is associated closely with advancing age [4,5,6,7]. In the last 20 years, a man’s lifetime risk of being diagnosed with PCa has increased considerably, and this is attributed largely to the introduction of PSA screening in the early 1990s [8,9]. On the other hand, the number of men dying from PCa has decreased over the same 20 year period as the disease can now be treated more effectively through a wide range of treatment options and at an earlier stage [10]. When a man is diagnosed with PCa, his concerns are likely to include; (i) will I survive? (ii) how will I be treated? and (iii) what effect will treatment have on my lifestyle? From a clinical perspective, the main questions following a diagnosis of PCa are; do the prognostic data provide clear guidance of which treatment option(s) may be best for the individual patient? and, how does this align with the patient's individual circumstances, i.e., lifestyle, perception of risk, life aspirations and numerous other factors which are weighted differently for each man [11]. It is increasingly apparent that most men die with PCa and not because of it and for many men the disease is over-treated which is associated with adverse side effects that can be worse than the symptoms of the disease itself.

The key areas of importance in current PCa biomarker research are therefore to (i) achieve ‘improved’ diagnosis of PCa to identify those with life-threatening disease; (ii) identify additional biomarkers to guide the choice the treatment options most likely to be effective and (iii) establish biomarkers to identify disease recurrence/resistance to treatment as early as possible.

In this review, we discuss some of the main features of PCa that contribute to the difficulties in diagnosing and treating the disease which in turn create challenges in identifying biomarkers of clinical utility. Here, we provide a comprehensive overview of the application of proteomics to the discovery and development of protein biomarkers and their translation to clinical tests that may address the significant unmet needs in PCa diagnosis and treatment.

## 2. Prostate Cancer

Anatomically, the human prostate is made up of three morphologically distinct regions: the peripheral, transition and the central zones [12]. Different prostatic diseases may appear in each of these zones. Thus, benign prostatic hyperplasia (BPH) is a non-malignant growth, which commonly occurs in aging men and is generally found in the transition zone. PCa on the other hand is a malignant growth that arises primarily in the peripheral zone where it arises from the epithelial cells of the prostate gland and is therefore described as an adenocarcinoma [4,13,14,15]. Well-differentiated, low-grade tumors contain glandular structures containing tumor cells that express known PCa markers such as androgen receptor (AR) and PSA, while poorly differentiated PCa tumors are lacking in glandular structures and differentiated cells. As with many cancers, the cellular heterogeneity found in PCa represents a fundamental challenge to treatment and diagnosis. It is widely accepted that, through genetic mutation or otherwise, prostate tumors contain subpopulations of cells that are resistant to therapeutic intervention and give rise to cells of metastatic potential [16]. Notably, it has been demonstrated that between patients and even within the same patient there are substantial differences in the genotype and phenotype of metastatic PCa cells [16,17] and that the genetic heterogeneity of metastases can be traced to the primary carcinoma. Deep molecular profiling has also uncovered a substantial degree of heterogeneity and the existence of diverse cancer clones within an individual’s primary tumor [18]. It is evident therefore that PCa may develop from multiple clones which give rise to a single PCa mass [19]. Although significant effort has gone into identifying the common genetic aberrations that contribute to the mutational landscape of PCa, deriving any therapeutic benefit from this information is impeded by the high levels of intra-tumoral heterogeneity as well as the long natural history from diagnosis to metastases or lethality [20,21]. So, although not associated with a high mortality rate, PCa is a complex malignancy that still needs to be understood more thoroughly at a molecular level to improve on current diagnostic and treatment strategies.

### 2.1. PSA Screening and Prostate Cancer Diagnosis

**Figure 1:**
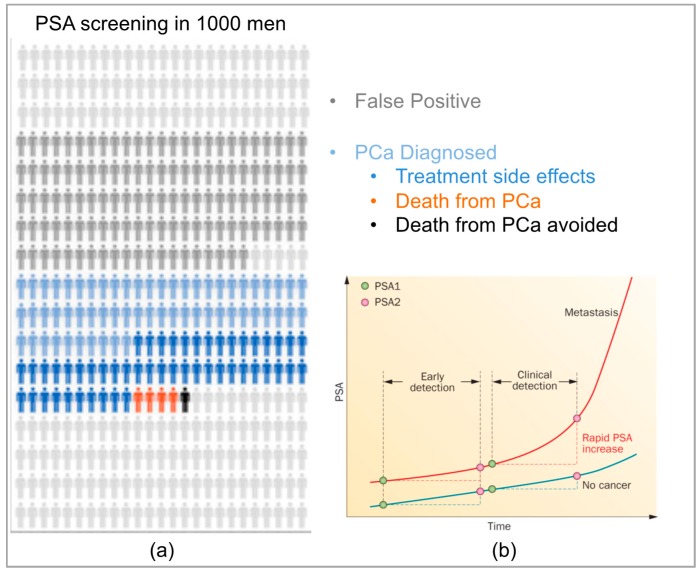
PSA screening and over diagnosis: The info graphic indicates the proportion of patients for which a true diagnosis versus of prostate cancer is achieved as result of PSA screening (**a**); A common reason for misdiagnosis is that a similar trajectory of PSA increase is often observed in men who have benign prostatic hyperplasia (BPH) (**b**). Figure adapted from Lin, K et al. and Roobol, M., et al. (2012) [30,31].

The goal of an ideal cancer screening approach is to identify individuals within a population who have cancer and, where possible, to select those for whom it is appropriate to intervene therapeutically [1]. Since it was first described as a prostate specific protein and a potential marker for PCa, the level of PSA (prostate specific antigen) protein in blood has become the most commonly used molecular marker for screening, diagnosis and management of PCa and indeed is the most widely used screening marker for any cancer [22]. PSA is a 30–33 kDa protein that is secreted into the seminal fluid by luminal epithelial cells of the ducts and acini in the prostate. PSA is limited from release into the circulation (blood) by the normal basement membrane of prostatic ducts and acini as well as prostatic stroma. Although associated with PCa, increased PSA levels can also be caused by benign events such as prostatitis and BPH (Figure 1) [23]. Furthermore, the level of PSA in blood, although correlated with long-term clinical outcomes at a population level, does not allow distinction between benign and aggressive PCa in individuals. Elevated levels of PSA generally lead to a subsequent digital rectal exam and tissue biopsy to confirm the diagnosis (and stage) of PCa. Notably, the prediction of disease outcome based on PSA levels alone is highly uncertain [24,25] and the value of PSA screening is almost obsolete for rapidly progressing cancers, which are in a pre-clinical state for a much shorter period of time, and are usually undetectable at an early stage [25]. Moreover, the relationship between PSA and chances of a positive biopsy diagnosis is also unclear. In a retrospective study using data from five European and three US cohorts of men, it was found that the association between PSA and positive biopsy was widely varied in terms of both the probability of PCa at a given PSA value and the shape of the risk curve, which is used to provide prediction between low grade (Gleason < 7) and high grade PCa [26]. Screening for PSA is sufficiently sensitive to detect many low-risk cancers and as such has also been associated with a large increase in the number of men over-diagnosed and over-treated for PCa [1,27]. Indeed, a study has shown that the extent of over-diagnosis associated with PSA screening is estimated to be as high as 25%–50% [28] (Figure 1). Not surprisingly, appropriate implementation of PSA screening is a highly debated subject. Although it is now recognized to have many limitations, the use of PSA has contributed to a paradigm for the value of protein biomarkers in detecting disease. The main weakness of the PSA assay is in its specificity, which researchers believe can be improved by the incorporation of additional protein biomarkers to create a multiplexed protein panel [10,29].

### 2.2. Disease Stratification and Curative Treatment Strategies

There are numerous treatment options available for men diagnosed with PCa, dependent on the severity of disease. The main indication of disease risk is determined by the Gleason score which is based on the glandular architecture of various areas of the tumor specimen (biopsy sample), observed at low magnification [20]. The Gleason grading system uses five basic grades (based on observed tumor growth patterns) which are used to generate a histologic score ranging from 2 to 10, by adding the primary grade pattern to the secondary grade pattern [32]. Although this grading system has been used since the 1970s, it is now accepted that the original assignment of cancer stage based on Gleason score is not appropriate for accurate staging of PCa tumors. This is largely due to the ambiguity surrounding a Gleason score of 7. Many studies have shown that patient outcomes will vary based on whether their Gleason score of 7 represents a tumor that is mostly Gleason 4 with some Gleason 3, or vice versa [33,34,35]. A modified version of the Gleason scoring system has therefore been introduced in which PCa tumors are graded as follows: grade group 1 (Gleason ≤ 6), grade group 2 (Gleason 3 + 4), grade group 3 (Gleason 4 + 3), grade group 4 (Gleason 8) and grade group 5 (Gleason 9–10). This revision of the Gleason scoring system has reportedly resulted in a more accurate grading system for PCa patients and provides a much better prognosis for patients diagnosed with Gleason 6 PCa [36,37].

Levels of PSA and Gleason score can be combined to classify patients according to their level of disease risk and thereby assist in determining the most appropriate treatment option [7,38]. Treatment options for early-stage PCa include observation/active surveillance, hormone therapy, radical prostatectomy and radiotherapy (Table 1) [39]. Of these, radical prostatectomy and radiotherapy are the two main first-line treatment options for organ confined PCa [10,39]. Approximately one third of patients diagnosed with PCa undergo radical prostatectomy in the early stages of their disease [40,41]. Radical prostatectomy is an effective treatment option for patients with localized, low to intermediate grade (Gleason score between 5 and 7) PCa and has been demonstrated to reduce the risk of death from the disease. Radiotherapy is also used as a main treatment modality in men with PCa. It can be included as an alternative to surgery although it is more often administered post-operatively, either alone or in combination with hormone therapy (CHRT)—depending on the stage of disease or the patient’s preference [42]. Hormone (androgen) ablation therapy is the primary treatment for metastatic (non-localized) PCa [1,43]. The idea behind androgen ablation therapy is to reduce levels of testosterone to castrate levels (<15 ng/dL), thus depriving the prostate cells of their most important stimulant for growth, function and proliferation [44]. Until recently, indefinite administration of mono- or combined androgen ablation therapy was the standard approach for treatment of advanced or metastatic PCa [45]. In recent years, a wide variety of novel therapeutic options have become available for advanced PCa [44,45,46]. Various combinations of established chemotherapy such as Docetaxel and novel androgen axis targeted agents such as Enzalutamide and Abiraterone with androgen ablation therapy or, indeed, the combination of hormone therapy with radiotherapy (CHRT) have shown the most promise in terms of decreasing PCa-associated mortality [47]. However, despite the reported efficacy of these treatments in management of PCa, they can take a considerable toll on a man’s quality of life. For a disease with which a man can expect to live for a considerable number of years, this is an important consideration when selecting an appropriate treatment option.

### 2.3. Impact of Curative Treatment on Patient Lifestyle

The main function of the prostate is to secrete an alkaline fluid containing protein which aids in motility, nourishment and protection for the sperm [6]. Despite this function, the prostate is not necessarily required for fertility and removal of the prostate has therefore been broadly considered as a relatively safe treatment option for men diagnosed with PCa. This view is supported by the low mortality rate of less than 0.3% for men with intermediate to high-risk PCa who are treated by radical prostatectomy. Indeed, some clinicians have advocated radical prostatectomy for low-risk patients with localized disease [48]. In reality, however, the procedure is associated with significant side effects that can and often do impact negatively on a man’s quality of life. These common side effects include; impotence, orgasmic dysfunction, incontinence, pulmonary emobolism, rectal injury, urethral strictures and the need for transfusion [49]. More than 50% of men are at risk for ejaculatory dysfunction, which has been cited as the primary concern of men receiving treatment for PCa [50]. Treatment of PCa is further complicated by a number of patient-specific compounding factors and co-morbidities that are associated with increased age. These include complications arising from cardiovascular disease and diabetes mellitus. In the past, radical prostatectomy, although a relatively straightforward procedure, would not have been considered for men aged ≥70 years who would have been presumed to have a life expectancy of less than 10 years. Currently, with increased life expectancy and the advent of minimally invasive surgical techniques, this is often no longer the case. However, the outcomes for older men who undergo radical prostatectomy are not as promising as compared to younger patients (<60 years old) [51,52]. Unfortunately, the risk to a man’s sexual function is also prevalent as a consequence of androgen deprivation therapy (ADT) [53]. Additional side effects to ADT include; decreased libido, osteoporosis, vasomotor flushing, fatigue, anaemia, diabetes mellitus, metabolic syndrome and altered body composition [54]. Again, a patient’s age has been shown to correlate significantly with the degree by which a patient will be affected by these side effects [54]. As well as age, other confounding factors in relation to sexually related side effects include the patient’s level of pretreatment function and drive, his degree of functional impairment and his sexual partner. Therefore, due to the chronic nature of PCa and the long period of time before the cancer evolves from a premalignant lesion to a clinically relevant cancer, treatment should be focused on the quality of life and sexual health of the patient as well as survival [50,51,54].

### 2.4. Impact of Curative Treatment on Patient Lifestyle

Active surveillance (AS) has become an alternative to curative therapy for patients that are deemed unlikely to develop biologically or clinically significant PCa [55]. The idea behind implementing AS is to prevent the overtreatment of patients with radical prostatectomy and/or hormone deprivation therapy. Ideally, patients who present with low-risk PCa would instead be monitored closely over time, without treatment. With a blood-based protein biomarker assay, any signs of more aggressive disease would be detected at a sufficiently early stage for curative treatment, thereby allowing the majority of patients to retain their current quality of life until treatment is absolutely necessary [56]. In 2006, the Prostate Cancer Research International Active Surveillance (PRIAS) study was initiated to assess the utility of AS in counteracting overtreatment in PCa. In their most recent report, in which 2492 ‘low risk’ PCa patients were followed for approximately 1.6 years, they suggest that AS is a feasible strategy to reduce overtreatment, although their follow-up was too short to draw definitive conclusions about the safety of AS [57]. A worldwide consensus for the appropriate criteria and protocols for AS has yet to be established. Only two organizations—the South East Scotland Cancer Network (SCAN) and Cancer Care Ontario (CCO)—have published guidelines that are specifically focused on AS, as opposed to most others that only offer information on AS as an alternative management option [58,59]. To address this lack of consensus, the Movember Global Action Plan 3 (GAP3) Active Surveillance project was initiated in August 2014. This 30-month initiative will allow creation of the largest centralized PCa AS database to date and will compromise the majority of the world’s AS patient data. The GAP3 project is being implemented across 19 institutions across 14 countries in five Movember regions (Australasia, Europe, UK, Canada, USA) as well as being open to other eligible centers. The overall aim is to provide and manage a worldwide platform with information and guidelines on AS as an accepted treatment option for PCa and to also reduce the number of men switching to active therapy within 1 year of starting the AS protocol [56,60]. To complement this, another of the Movember Global Action Plans (GAP1) is focused on the identification and validation of protein biomarkers that can more accurately distinguish between low-risk and aggressive forms of PCa and are measurable in blood, urine and tissue. The GAP1 project is, again, an international effort involving 50 principal investigators across 14 countries, with collaborators employing a number of the proteomic techniques that will be described in this review.

### 2.5. Clinical Need for Additional PCa Biomarkers

Although the majority of men diagnosed with localized PCa have indolent disease that will likely not be threatening to their expected lifespan, approximately 15%–25% of men who undergo curative treatment for localized disease will suffer cancer recurrence [15,38,61]. Moreover, 80%–90% of patients who receive androgen ablation therapy develop castration-resistant tumors within 12–33 months, for which there is no cure [43,62]. The earliest indication of PCa recurrence (treatment failure) is termed ‘biochemical recurrence’ and is diagnosed following two successive PSA measurements 2 ng above the nadir—the nadir value being the baseline PSA measurement for a patient immediately following radical prostatectomy [23,29,63]. However, not all patients with detectable PSA post-surgery will manifest clinical progression and some patients may suffer cancer recurrence without a pre-emptive increase in serum PSA [29]. This has been reflected in numerous studies, including a recent publication by Ehdaie et al which demonstrated significant variability in longitudinal measurements of PSA in individual PCa patients [8]. Despite much research into the area, few studies have been able to clearly identify parameters that can be used to more reliably predict local recurrence and thereby identify those patients who are more likely to benefit from treatment [64].

Using blood-based biomarkers to guide a biologically individualized approach to treatment would be much more ideal for both patients and clinicians and could improve treatment outcomes and survival rates by up to 10% [65]. Initially this was addressed by attempting to identify and measure additional iso-forms of PSA. Free PSA (fPSA) is the small amount of PSA that is not bound to serum proteins and the percentage of fPSA has been used to stratify the risk of PCa in men with total PSA levels of 4–10 ng/mL and a negative digital rectal exam (DRE). A meta-analysis has shown that the use of %fPSA improves the diagnostic performance among men with total PSA in the range of 2–10 ng/mL, compared with total PSA alone [66]. However, fPSA is unstable at 4 °C or room temperature and can produce conflicting results in men with BPH and prostatitis [67]. Both PSA velocity (PSAV) and PSA doubling time (PSADT) have been used to measure the change in PSA per year and specific value increases in PSA, respectively. These measurements are also considered to increase the specificity of PSA [67]. An isoform of proenzyme PSA called [-2] proenzyme PSA (p2PSA) has also been advocated as a target that can enhance the specificity of PSA-based screening [68]. In a prospective, population based study of 769 biopsied men aged <65, it was shown that p2PSA combined with the Prostate Health Index (PHI) score has a superior diagnostic performance for detection of PCa when serum PSA is in the range of 1.6–8.0 ng/mL [69]. In another model proposed by Grönberg et al., it was shown that a combination of plasma protein biomarkers (PSA, fPSA, hexokinase 2 (hK2), microeminoprotein beta (MSMB), macrophage inhibitory cytokine 1 (MIC1)), genetic polymorphisms and clinical variables (age, family history, previous prostate biopsy and prostate exam) performed significantly better than PSA alone for detection of cancers with a Gleason score of at least 7. Indeed it was proposed that this model—the STHLM3 model—could lead to reduced PCa mortality with substantially fewer biopsies and reduced over diagnosis [70]. These studies have highlighted the weaknesses of PSA as a single biomarker and further corroborate the need to identify multiple blood-based proteins that would be able to stratify PCa patients at critical stages throughout the disease.

In summary, appropriate management of PCa is made extremely difficult to the inherent heterogeneity of the disease and the weaknesses of PSA, which is still relied on quite heavily to monitor progression of PCa. Current treatment options for PCa, although effective, are associated with significant side effects and can have a detrimental effect on the quality of life for PCa patients. Although active surveillance is being increasingly advocated for the management of PCa, both patients and physicians are uncomfortable with forgoing immediate treatment. Ultimately, this is because existing PCa treatment decision tools do not specifically address issues relevant to low-risk PCa [71]. To this end, protein biomarkers that could accurately identify those patients at high risk for aggressive PCa, could prevent the over-treatment of those with low-risk disease, thereby preserving their quality of life.

## 3. Newly Emerging Tests for Prostate Cancer

### 3.1. Tissue Based Prostate Cancer Biomarkers

It is widely agreed that better understanding of PCa biology using tissue-based biomarkers might help clinicians to make more personalized treatment decisions [72]. To this end, the prognostic value of the protein Ki-67 has been well documented. This tissue-based marker has been shown to be a significant determinant of distant metastasis and PCa-related death [73,74,75]. In addition, phosphatase and tensin homologue (PTEN) loss has also been found to add prognostic value to Gleason score, PSA and Ki-67 tissue staining [76]. It has been shown that PTEN loss is routinely observed in prostate tumors with high Gleason grade, although it is recommended that it would only be of real use as a biomarker if combined with a panel of additional markers. Indeed, no CE-IVD level standardized assays exist for either PTEN or ki-67 currently [77].

With a view to establishing an assay based on a panel of PCa-specific markers, a number of tests have emerged which claim to better predict PCa occurrence based on the observed expression of multiple genes/proteins. One example is the Decipher test offered by Genome Dx Biosciences. This is a 22-marker genomic classifier containing a large number of non-coding RNA sequences that was both developed and verified in FFPE tumor tissue specimens. This test uses whole-transcriptome microarray assay for analysis of gene activity in FFPE PCa specimens [78]. The expression of these gene markers is used to calculate the probability of clinical metastasis within 5 years after radical prostatectomy, and within 3 years of biochemical recurrence [79]. The test can also offer risk assessment to help tailor treatment options for patients diagnosed with localized prostate cancer on biopsy. It has been reported that the Decipher test is superior in predicting early clinical metastasis when compared to previously described individual gene markers, multi-gene signatures and other clinicopathologic variables [80]. Additionally, in a clinical utility study conducted by Badani et al., it was found that the information provided by this test does influence the judgment of urologists in selecting an appropriate secondary therapy in both adjuvant and salvage settings [78].

A similar test—the OncotypeDX offered by Genomic Health Inc. (Redwood City, CA, USA)—also measures a 17-gene signature as an independent predictor of adverse pathology in PCa. The signature is comprised of 5 reference genes (for normalization) and 12 cancer genes which represent biological pathways with a known role in PCa progression; the androgen pathway, cellular organization pathway, proliferation pathway and stromal response pathway [81]. This test was developed in a bid to address the impact of tumor sampling in predicting aggressive PCa, i.e., by overcoming the inherent genetic variations between regions of individual tumors and the limited tumor material acquired by needle biopsy [82]. The RT-PCR-based assay has been clinically validated to predict the risk of high grade and/or non-organ confined PCa at radical prostatectomy using biopsy samples containing as little as 1 mm of tumor tissue [79,81].

Recently, a test based on the expression of cell cycle progression genes in primary tumor samples has shown great promise in accurately stratifying patients with localized PCa according to disease aggressiveness. The ‘Prolaris’ test, which is offered by Myriad Genetics Inc. (Salt Lake City, UT, USA), is a genomic test for predicting PCa aggressiveness in conjunction with clinical parameters such as Gleason Score and PSA [79]. This RNA expression-based assay directly measures tumor cell growth characteristics. The test combines the gene expression levels of 31 cell cycle progression (CPP) genes and 15 house-keeping genes to give a CPP score [83]. The CPP signature was originally validated in both a retrospective cohort (336 patients) from the US who had undergone radical prostatectomy and a UK cohort of 337 patients with clinically localized PCa diagnosed by transurethral resection (TRUP) [84]. In a univariate analysis it was found that the CPP score alone could accurately predict biochemical recurrence. In a multivariate analysis, where CPP was combined with additional clinical parameters for PCa diagnosis, it was found that CPP and PSA were the most significant predictors of recurrence and provided much more prognostic information than any other variable in both cohorts. This assay has since been validated in numerous cohorts representing disparate patient populations using both tumor biopsy samples and paraffin sections [85,86,87,88]. Currently, it is envisaged that this test will be most applicable in helping to identify low-risk patients who can be safely managed with active surveillance [38]. Results of a meta-analysis of five studies were presented at AUA 2016 and show the test to be a significant predictor of oncologic outcomes in patient with low risk disease defined by Gleason Score <7 [89]. Further validation of the Prolaris assay is underway in larger, community-based cohorts with more results anticipated for later in 2016.

Metamark offer the ‘ProMark’ assay, which is a protein based prognostic test for predicting PCa aggressiveness—particularly for patients with Gleason grade 7 disease [79]. This assay was originally designed to measure 12 protein markers in patient biopsy specimens [72]. The signature has since been refined to eight protein markers which are predictive of PCa aggressiveness and measured using a multiplexed in situ imaging system [90]. The test has been shown to reproducibly provide simultaneous quantification of protein levels and functional activities using tissue specimens [91]. The intended use of this test is to supplement current biopsy-based PCa risk assessment methods in cases where a clinical decision regarding active surveillance versus active treatment is not straightforward. PCa is a highly heterogeneous and multifocal disease and so, the eight biomarkers which comprise the ProMark assay have been specifically selected and evaluated to predict pathology outcome whether they are measured in low or high grade tumor specimens from the same patient. As such, the ProMark test can perform accurately and with high sensitivity, even in tissue samples with variable amounts of tumor versus benign components. Indeed, the ProMark assay has been validated in an independent blinded study and shown to complement current risk stratification systems [92].

Although the tests described here show a great deal of utility in stratifying patients for appropriate PCa treatment, they are all dependent on the availability of tumor tissue samples. Therefore, these tests are faced with one of the main limitations for development of routinely used and robust clinical assays, namely that biopsy-based analysis is generally associated with at least a 20% error rate due to (a) the tissue sampling error associated the limited amount of sample acquired during biopsy; (b) the tissue heterogeneity of the disease and; (c) the complex procedures implemented to preserve tissue samples. Furthermore, there is little to no overlap in the genes/proteins analyzed in the different tests and, perhaps not surprisingly, their overall utility remains unknown. To overcome many of these limitations, it would be highly desirable to have a clinical test based on gene/protein measurement in a patient biofluid which is less limited in sample access, obtained less invasively, easier to sample repeatedly and can be processed for storage and analysis in a more standardized and less complex manner.

### 3.2. Fluid Based Prostate Cancer Biomarkers

There has been a significant investment in the discovery of gene and protein biomarkers and the development of biofluid based assays for improved diagnosis and treatment of PCa patients. Gene-based assays have, to date, made much more progress than proteomic-based assays. As an example, the expression of a gene called DD3^PCA3^, which codes for a protein called Prostate Cancer Antigen 3 (PCA3), has been shown to correlate with malignant PCa. Indeed, it has been demonstrated that PCA3 is not at all expressed in normal prostate tissue and expressed at very low levels in BPH specimens [93]. Moreover, the expression of PCA3 can be measured in urine. The Progensa assay compares the concentration of PCA3 mRNA levels to PSA mRNA levels to produce a urinary PCA3 score [94] and it has been found that urinary PCA3 scores (PCA3-mRNA/PSA-mRNA) are consistently superior to serum PSA levels for diagnosis of PCa. Unlike PSA, PCA3 expression remains constant during prostatic hyperplasia and prostatitis, thereby making it more sensitive than PSA for PCa detection and it has therefore been suggested that the PCA3 score be used as an exclusion tool [95,96]. The main downside to this test, however, is that it can only be performed using the first 20–30 mL of urine voided after a digital rectal exam (DRE) so it only provides valid results in approximately 80% of cases [95]. The measurement of PCA3 has also been combined with another well-known biomarker of PCa—the *TMPRSS2:ERG* gene fusion as part of the Mi-Prostate Score [97]. Both the PCA3 and *TMPRSS2:ERG* biomarkers can be detected in patient’s urine after DRE which provides a basis for a non-invasive, easy to use clinical test. This validated test, which is offered by the University of Michigan MLabs incorporates blood PSA levels with urinary levels of PCA3 and *TMPRSS2:ERG* to allow for stratification of PCa while avoiding unnecessary biopsies [79,97,98].

A newly available urine test from the same team who developed the PCA3 test and offered by MDx Health is SelectMDx, which measures expression of HOXC6 and DLX1 genes in urine using KLK3 (PSA) used as an internal reference. This test was designed following a study by Leyton et al., which identified 39 PCa biomarkers from gene expression profiling data. Quantitative PCR analysis on both tissue and urine samples led to the identification of 8 urinary biomarkers for PCa which was subsequently refined to a 3-gene panel—HOXC6, TDRD1 and DLX1. This urinary 3-gene panel showed higher accuracy in detecting aggressive (Gleason > 7) PCa compared to the Progensa PCA3 assay [99]. Subsequently, two prospective multicenter studies were conducted to validate the gene panel based on whole urine and develop a model combining molecular profiling with traditional clinical risk factors. The risk score derived from combining the two most promising gene markers—HOXC6 and DLX1—with PSAD, DRE and PSA was found give the most accurate detection of high grade PCa on biopsy and was also successfully validated in an independent patient cohort [100].

PCA3 has also been incorporated into a new test called the ExoDx Prostate Intelliscore, which is offered by ExosomeDx. This test measures PCA3 along with two other exosomal RNAs which are known to be expressed in men with high grade PCa. Using a proprietary algorithm integrating the hree genes with standard of care measurements the test can predict whether patients presenting for initial biopsy are have aggressive disease with an AUC of 0.73 (95% CI = 0.68–0.77) [101]. The ExoDx Prostate test aims to reduce the number of unnecessary biopsies and will be available in the US this year as a Clinical Laboratory Improvement Amendments (CLIA)-based clinical laboratory-developed test (LDT).

Another urine test called Prostarix (Metabolon Inc. Durham, NC, USA) uses metabolomics technology to measure levels of four amino acids associated with PCa. Using liquid chromatography and mass spectrometry coupled with a logistic regression algorithm to generate a score, the test claims to aid the assessment of cancer detection and can be used to distinguish between benign prostate, clinically localized PCa and metastatic disease [102].

The recent successes in clinical research on serum-based biomarkers for PCa detection remain confined to the kallikrein field [103]. A four prostate-specific kallikrein panel has shown great promise as a serum-based test for PCa. The 4Kscore is a combined measurement of total PSA, fPSA, intact PSA and human kallikrein-related peptide 2 (hK2). It has been observed in multiple studies that the serum 4Kscore assay accurately predicts the risk of biopsy-detectable high-grade PCa in men who have not undergone a prostate biopsy [104]. Indeed, one study showed it to be more predictive of PCa than PCA3, and it was therefore recommended for use alongside PCA3 for detection of PCa in pre-screened men [105]. The 4Kscore is now commercially available in the US as a CLIA-approved LDT. Unlike PSA, however, the 4kscore is not (currently) FDA approved although it appears to have some clinical utility [94].

The PCa tests described here are summarized in Table 2. Although these tests are certainly promising, due to their novelty longitudinal studies addressing their clinical benefit when implemented outside of tightly controlled studies are warranted [106]. Ultimately, to integrate a new marker into clinical decision making, it must prove to be superior to the standard measures currently in use [107]. Another challenge to overcome with regards to the urine and serum-based tests is the dilution effect of measuring secreted genes/protein in bodily fluids based on which tumor/tissue regions they originate from. In this respect, the choice of technology with which to measure markers of interest will be a key consideration for the successful development and clinical implementation of fluid-based PCa biomarkers.

## 4. Proteomics to Answer Key Questions in Prostate Cancer

**Figure 2:**
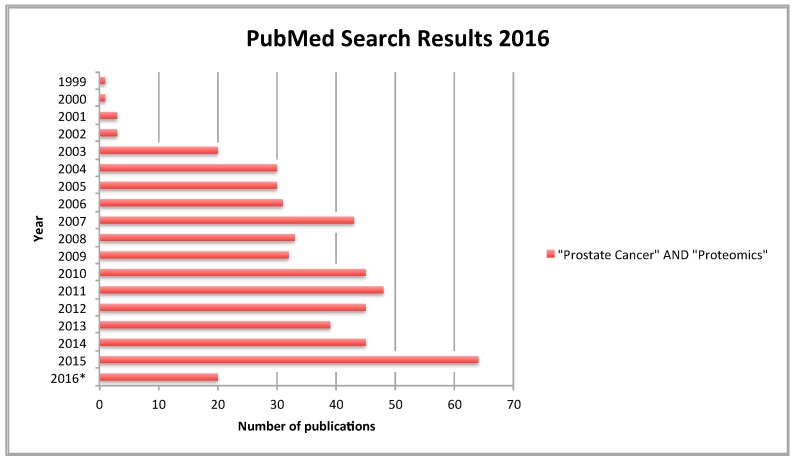
PubMed Search Results for Proteomics and Prostate Cancer: A PubMed search was conducted in March 2016 with the search terms “Prostate Cancer” AND “Proteomics”. The total number of ‘hits’ was 533, with dramatic increases observed for the years 2003 and 2013.

The term proteomics was introduced as an analogy to that of ‘genomics’ [108]. While genomics involves the study of the genes that code for a protein, proteomics is focused on studying the proteins themselves—thus providing a clearer reflection on cellular activity [109]. Proteomic-based experiments can be used to characterize any alterations in protein expression during disease progression [110,111]. The emerging field of proteomics has had a tangible impact on biomarker discovery in PCa (Figure 2). A useful cancer protein biomarker would be a protein measurable in body fluids or tissues that could reflect the presence of cancer and provide information on the cancer’s stage, aggressiveness and how well the patient is responding to therapy [112]. For such a biomarker to be clinically applicable, however, it must also meet the following criteria: (i) the protein must be easy to measure at a reasonable cost; (ii) elevated levels of the protein must provide information that would not be available without that protein and (iii) the information obtained from measurement of the protein can be used to guide clinical decision making [113,114]. Due to the complex nature of cancer, uniformity is non-existent among each histologic cancer type and within each individual tumor. As such, examination of combinations of potential protein biomarkers as panels is believed to provide greater promise for improved PCa diagnosis and monitoring [115]. This trend is reflected in the most recent publications related to PCa associated biomarker discovery (Table 3).

According to Rifai et al., the process of identifying new protein biomarkers is conducted in four main stages, beginning with an initial discovery phase and ending with a final evaluation phase [116]. This process requires technologies that will allow for fast and consistent identification of proteins spanning the expansive dynamic range of the disease proteome [111]. For the initial discovery phase, those proteins, which appear to be changing as result of disease activity, can be identified in any biological sample; however, there are advantages and disadvantages associated with biomarker discovery in the various biological samples used (Table 4).

## 5. Biological Sources for Biomarker Discovery in Prostate Cancer

### 5.1. Tissue

Tissue and cell culture models are appealing for biomarker discovery in that they can be directly manipulated to investigate the expression and/or role of certain proteins, as direct result of drug treatment or viral infection [144]. Furthermore, tissue and cell culturing allows for analysis of single cell populations (e.g., fibroblasts or macrophages) should they be of particular interest, as well as mixed cell populations, similar to what would be found in the in vivo environment [145]. The main downside to identifying protein biomarkers in this manner, of course, is that tissue and cells cultured outside of the human body cannot provide accurate insight into disease progression in vivo. Aside from culturing of tissue cells, proteomic experiments can also be performed on fresh tissue specimens. This method is slightly more challenging as harvesting and processing tissue specimens must be performed as quickly as possible to avoid any protein degradation [146]. To preserve their proteome integrity, tissue samples can be snap frozen or fixed using formalin and embedded in paraffin wax. The latter is referred to as formalin fixed paraffin embedded (FFPE) tissue, and this is the universal method for tissue preservation and stabilization [147]. In the field of proteomics, patient tissue samples are most commonly used to validate the expression of proteins of interest. However, in recent years numerous protocols have been optimized for efficient protein extraction of FFPE material for subsequent proteomic analysis via both antibody and mass spectrometry-based techniques [148,149]. Techniques for harvesting cells directly from tissue samples have also evolved in the last number of years. For example, coupling Laser capture microdissection (LCM) with proteomics allows for the analysis of proteins from specified regions of interest within a tissue section. This approach has been successfully applied for the proteomic characterization of regions of Gleason 3 and Gleason 4 PCa tumor tissue [150,151,152].

### 5.2. Blood

**Figure 3:**
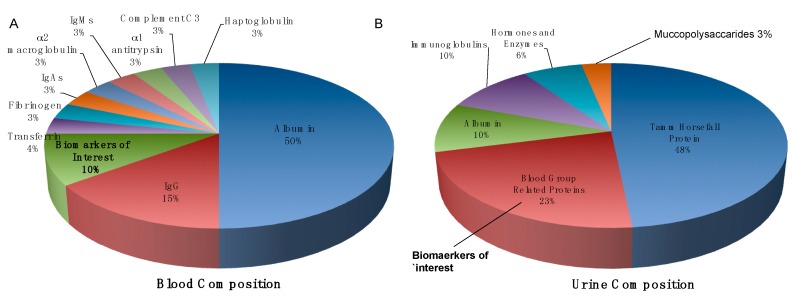
Dynamic range of protein concentrations in biological fluids: Figure 3 gives a breakdown of the concentration of proteins found in blood (**A**) and urine (**B**) samples. Notably, blood proteins, which are considered to be significant to biomarker discovery studies, make up less than 10% of the total concentration of blood proteins [167,170].

Blood circulates through the entire human body and contains proteins that are secreted by all cells and tissues. Therefore, even though the most prominent molecular changes will occur at the site of tumor formation and in surrounding tissue, such changes are likely to be reflected in the blood also. Whole blood is made up of serum, plasma, red blood cells (RBCs), white blood cells (WBCs) and clotting factors. Of these, serum and plasma are the fractions most often used for routine blood testing in hospitals and clinics as they contain many proteins that are synthesized, secreted, shed or lost from the cells and tissues throughout the body [153,154]. Serum and plasma are very similar in composition, both containing components such as glucose, electrolytes, antibodies, antigens, hormones, proteins, enzymes, nutrients and other small molecules. Serum is obtained from coagulated blood—fibrin clots formed during coagulation along with blood cells and related coagulation factors are separated from serum by centrifugation. Plasma, on the other hand, contains clotting factors and must be treated with an anti-coagulant such as EDTA or heparin before the removal of blood cells [155]. Serum and plasma represent readily accessible and clinically relevant samples for biomarker discovery and validation [156,157,158,159]. However, there are certain *caveats* to be considered when working with serum and plasma in the context of biomarker discovery. Sample processing and storage conditions must be conducted under standard operating procedures to ensure reproducibility in the data obtained across various laboratories [160]. This is an important factor that is often not considered. The main issue with regards to serum/plasma proteomics, however, is the expansive dynamic range of the proteome, which spans over 12 orders of magnitude [161]. In fact, the majority (95%) of serum or plasma proteins are made up of a few high abundant proteins such as albumin, immunoglobulins, alpha-1-antitrypsin and haptoglobins, etc., which can mask the presence of potentially significant low abundant proteins (Figure 3) [156]. To overcome this, numerous fractionation, depletion and enrichment techniques are often implemented to enhance the detectability of low abundant proteins. Although this is a useful strategy for proteomics discovery, when it comes to validation of potential protein biomarkers, the additional sample preparation techniques can introduce considerable variability, as well as adding to the time and cost of running a blood-based diagnostic assay.

### 5.3. Urine

Urine is considered to be the ultrafiltrate of blood and is a popular biofluid for diagnostics and biomarker discovery [162,163]. Urine is favored as a source for biomarkers for several reasons; (i) large quantities of urine can be obtained non-invasively and trained personal are not required to obtain a sample; (ii) urine contains proteins and peptides of low molecular weight that can be analyzed without excessive sample preparation and (iii) urine is a highly stable body fluid. It is stored for hours in the bladder and therefore any proteolytic degradation is essentially complete by the time urine is voided [164]. Furthermore, with regards to PCa, fluids that are proximal to the prostate are attractive sources of potential biomarkers as they are likely to contain secreted proteins that can be used to assess the extent of disease [165,166]. Again, however, there are a number of caveats with regards to using urine as a biological source for biomarker discovery; (i) as samples are not collected by trained personnel, it is difficult to control for variability in sample collection; (ii) much like plasma and serum, the protein composition of urine spans a wide concentration range (Figure 3) and (iii) urine composition is greatly influenced by patient-related variances such as collection time, diet, exercise and disease stage [164]. Despite these pitfalls, however, urine has been described as the next frontier for biomarker development for PCa [167]. Indeed, proteomic studies of urine have been successfully implemented for the discovery of novel biomarkers for diagnosis, surveillance and monitoring of disease progression, as demonstrated by the success of the Progensa (PCA3) assay [96]. Many researchers have also successfully identified and measured additional potential PCa biomarkers in urine. For example, in a study conducted by Jedinak et al. patient urine samples were used for the identification and validation of urinary biomarkers that would distinguish between BPH and localized PCa. Here, a panel of three urinary protein biomarkers, β2M, PGA3 and MUC3, were found to effectively discriminate between BPH and localized Pca [168]. In a similar study conducted by Davalieva et al., a panel of urinary proteins including; Annexin A3, Inter-alph-trypsin Inhibitor Heavy Chain 4, CD90, Calgranulin/MRP-14, Semenogelin 1, Uromodulin and Engrailed-2 were found to show significantly differentiated expression between patients with BPH and patients with localized PCa [166]. Another protein, which is commonly associated with PCa, zinc alpha 2-glycoprotein (ZAG), has also been successfully measured and evaluated in urine samples from PCa patients [169].

### 5.4. Semen

Seminal fluid is produced by male accessory sexual glands including the prostate, seminal vesicles, epididymis and Cowper’s gland. A multi composition of seminal fluid includes acid phosphatase, inositol, citric acid, calcium, magnesium, zinc, fructose, ascorbic acid, prostaglandins, l-carnitine and neutral alpha-glucosidase [171]. Moreover, seminal fluid contains high amounts of proteins and amino acids, ranging from 35 to 55 g/L, and is therefore is theoretically a good sample source for protein identification. For proteomics-based discovery experiments, an essential step in semen sample preparation is the purification of seminal fluid from sperm cells and any other semen containing cells. This can be achieved through density gradient centrifugation using commercially available kits such as PureSperm or Percoll. Alternatively, a method known as ‘swim-up’ has also been described [172]. Semen is considered a relevant biological source for investigations based on fertility and diseases such as PCa [173,174]. For example, to assess male fertility semen is often used to determine spermatozoa morphology, motility and concentration [175]. A systematic assessment of pre-analytical seminal plasma stability and it’s suitability for the development of PCa biomarkers was recently undertaken by Neuhaus et al [176]. Preliminary data from this study indicated that there was minimal post-sampling degradation, which is in contrast to what is often observed for blood serum or plasma. Moreover, with their relatively modest cohorts, Neuhaus et al. discovered a seminal biomarker signature that would distinguish patients with a post-surgery Gleason score of 7 as having either indolent or advanced PCa. The sensitivity and specificity observed for these markers was actually better than when the same markers were measured in urine [177]. Although these results are promising, access to seminal fluid is complicated by low patient compliance, social behavioral norms and ejaculatory dysfunction in the aged population—particularly in men with PCa. As with blood and urine, semen also contains a large proportion of high abundant proteins that can mask the lower abundant proteins and make them more difficult to detect through proteomic analysis. Furthermore, semen composition varies within individuals, between individuals and within a single ejaculate. Therefore, proteomics-based studies of seminal fluid can be challenging [178]. Expressed prostatic secretions (EPS) are considered a more accessible source for studies relating to PCa. EPS is collected just prior to prostatectomy from vigorous digital rectal massage, which forces prostatic fluids into the urethra for collection from the penis. One of the main advantages of this fluid as a biomarker source is that it provides clinical information that would have led to the decision to perform a prostatectomy in the first place. Also, prostate-specific proteins, such as PSA, are present in large amounts and therefore easily detectable by mass spectrometry [179]. This said it is unlikely that semen-based investigations will lead to the identification of novel biomarkers or that semen would be used for routine clinical screening tests.

In summary, there are multiple biologically relevant sources for the identification of clinically significant PCa biomarkers. The selection of an appropriate sample material for which to base initial discovery experiment will be influenced by the associated advantages and limitations which are outlined in the text and summarized in Table 4. The most clinically useful biomarkers are likely to be those that are measurable in blood or urine and so, use of these biofluids in biomarker related studies is probably the most desirable option. However, this will require the optimization of analytical techniques so as to overcome the main *caveats* associated with use of these samples—namely the dynamic concentration range of its protein composition, the low abundance of potentially significant protein biomarkers and the inter and intra-patient variability in composition (Table 4).

## 6. Proteomic Technology for Biomarker Discovery and Validation

### 6.1. Biomarker Discovery

#### 6.1.1. Gel-Based

Two-dimensional polyacrylamide gel electrophoresis (2D-PAGE) is one of the core technologies used in proteomics and was once considered the ‘state-of-the-art’ method for protein separation and expression profiling [180]. 2D-PAGE has been used since the 1970s and involves the separation of proteins according to their isoelectric point (on the *y*-axis) and their molecular weight (*x*-axis) by sodium dodecyl sulfate (SDS) electrophoresis. With this technique more than 1000 proteins can be detected and quantified [181]. However, accurate quantitative comparison between different gels, has been greatly impaired by gel-to-gel variation which led to inherently poor reproducibility in comparative proteomics [182,183]. To overcome this issue difference gel electrophoresis (DIGE) was developed with the premise that, by using labels that provide different fluorescence wavelengths for detection, multiple different samples can be combined and co-separated on the same gel [182]. Moreover, the technique also includes an internal standard within each gel to overcome the problem of integral variation [129]. In DIGE, samples are labeled with fluorescent CyDyes™ (Cy2, Cy3 and Cy5) prior to electrophoresis. The samples are then mixed before isoelectric focusing (IEF) and resolved simultaneously on the same gel [183]. For DIGE to be successful, the dyes must meet a certain set of criteria: (i) each set of matched dyes must react with the same amino acid residues; (ii) they must not change the charge of the target amino acid; (iii) they must be of similar molecular weight and (iv) they must have distinct fluorescent characteristics [184]. 2D-DIGE is now recognized as an accurate method to determine and quantify human protein expression [128]. With the advent of mass spectrometry for proteomics research, 2D-DIGE is generally coupled with down-stream mass spectrometry to identify protein spots, which show differential expression between the samples being analyzed. This is achieved by using gel imaging software to apply statistical analysis for detection of significant changes in protein expression between samples. Protein spots of interest are then simply excised from the gel and tryptically digested for MS analysis. This has become a widely used technique in research directed towards the identification of PCa biomarkers in patient samples [123,185,186]. In a study by Jiang et al., three proteins out of 60 were found to be differentially expressed between PCa and adjacent benign tissues using 2DE-DIGE (PTEN, SFPQ and HDAC1) were evaluated by both ELISA and immunohistochemistry and shown to have a significant association with PCa [128]. This technology has also successfully been applied for analysis of patient biofluids. In one study by Ummanni et al. analysis of urine samples from two age-matched groups of patients with histologically characterized diagnosis of PCa and BPH, led to the identification of 23 potential PCa biomarkers [139]. In another study by this group, 2DE-DIGE was applied to investigate serum autoantibody signatures for use as PCa specific biomarkers. Briefly, PCa proteins were resolved by 2DE-DIGE and transferred onto PVDE membranes that were incubated in pooled serum from either PCa or ‘healthy’ individuals. This resulted in the identification of 18 differentially expressed antigens. Two of these—PRDX6 and ANXA11—were able to discriminate between PCa and control patients with a sensitivity of 90% for PCa patients and 100% for healthy controls. The authors hypothesize that these serum autoantibodies could be used, in conjunction with established clinical tests, to aid diagnosis of PCa [187]. In another serum-based study Byrne et al. conducted a pilot 2DE-DIGE analyses on affinity-depleted serum in a bid to identify significant changes in lower abundant serum proteins. This study confirmed PCa-associated changes in the expression for PEDF and ZAG, not only in the original sample set, but also in an independent series of serum and tissue samples [186]. Although, as these studies have shown, 2DE-DIGE enables the separation of thousands of proteins, protein isoforms and protein modifications, the technology is unable to detect the presence of very large or small proteins, membrane-associated proteins, hydrophobic proteins and very basic or very acidic proteins—thus limiting the proteomic coverage of most biological samples [182,185].

#### 6.1.2. Mass Spectrometry-Based

Despite the technical advancements described thus far, mass spectrometry-based proteomic techniques remain at the forefront for the discovery and validation of blood-based biomarkers due to their ability to profile the complex proteomes of biological samples in an unbiased manner [188]. A mass spectrometer is made up of an ion source, a mass analyzer to measure the mass-to-charge ratio (*m*/*z*) of the ionized analytes, and a detector that registers the number of ions at each *m*/*z* value [189,190]. Over the last number of years, mass spectrometry has emerged as an invaluable technology for the quantification of thousands of proteins as well as their modifications, localization, turnover and interaction partners [191]. Shotgun or ‘bottom-up’ approaches are most commonly used in proteomics analysis. This approach involves proteolytic digestion of complex samples and analysis of the resulting peptide mixtures using liquid chromatography-tandem mass spectrometry (LC-MS/MS) in a data dependent acquisition mode [192]. Typically, peptides are separated based on their hydrophobicity and then electrosprayed into the mass spectrometer where they are sequenced by tandem mass spectrometry [193]. For the purposes of biomarker discovery, ‘hybrid’ instruments are widely used due to their unparalleled analytical specificity. Hybrid mass spectrometers typically refer to high-resolution instruments that are coupled to a front-end component that enables fragmentation of peptides (Q-ToF, Triple-TOF, Q-Orbitrap). These analyzers can now fragment several thousand peptides per hour [189]. Indeed it has been reported that over 70% of known proteins have been identified through mass spectrometry-based discovery experiments, with emphasis now shifting towards ‘deeper’ proteomic discovery experiments to detect the remaining 30%–35% of proteins [194]. To this end, one of the afore-mentioned hybrid instruments, the Triple-TOF, has enabled a new peptide detection strategy called ‘sequential windowed acquisition of all theoretical ions’ (SWATH), which attempts to analyze the fragmentation products of all ions generated during an analysis [195]. The premise of this technology is to combine the strength of regular shotgun proteomics with the reproducibility of MRM signaling to detect and quantify large numbers of analytes [196]. SWATH continuously fragments all peptides within stepped *m*/*z* windows. In a standard acquisition, 32 precursor windows of 25 Da in width are sequentially selected in the first quadrupole. In the second quadrupole, transmitted ions are fragmented and product ions are subsequently detected in the Time of Flight (ToF) mass analyzer. The resulting transition ions are matched to a spectral library for identification and quantification of proteins/peptides [197,198]. The SWATH method relies heavily on peptide spectral libraries, which must be established in advance through standard shotgun methods. The ability to simultaneously perform a large number of MRM-type assays makes this approach very promising for label-free quantification of panels of PCa biomarkers using minimal sample material [197,198,199]. SWATH technology has been applied for the identification and quantification of glycopeptides in tissue samples taken from PCa patients. The resulting dataset led to the identification of regulated proteins and pathways that have the potential to discriminate between patients with aggressive and non-aggressive PCa [200]. The technology has also been successfully applied in blood, with quantification of 342 plasma proteins across 232 plasma samples in a longitudinal study [201]. As this technology continuous to develop, it is anticipated that there will soon be MS spectral libraries to represent peptides covering the entirety of the human proteome that is currently available. It is therefore expected that, because of the unique ability to monitor all detectable protein species in a sample, with constant sensitivity and reproducibility across large sample cohorts, SWATH will become a more common feature in clinical research [202].

### 6.2. Biomarker Evaluation

#### 6.2.1. Antibody-Based

In the past, clinical evaluation of novel disease biomarkers has relied primarily on immunoassays due to their proposed specificity for the target analyte, sensitivity and high throughput [203]. For a long time, Enzyme Linked Immunoabsorbant Assay (ELISA) was the gold standard for protein detection in patient serum samples, having first been reported for use in verification studies in the 1970s. In a typical double antibody sandwich ELISA, an antibody attached to the bottom of a well provides both antigen capture and immune specificity while another antibody linked to an enzyme provides the detection and amplification factors for protein detection [204]. As multiplexed protein measurement has become of increasing interest—due to the lack of specificity observed for individual markers—the ELISA technique has been modified to allow for multiplexed measurement of protein biomarkers in a 96-well plate format. However, multiplexed ELISAs are expensive and time consuming, require large sample volumes with complicated dilution steps and only cover a narrow dynamic range of protein concentration. Many studies aimed towards the evaluation of potential PCa biomarkers have availed of this technique, however, a wide variety of variable factors are known to affect the performance characteristics of an ELISA. These include; the antibodies used, the temperature, the pH and the antibody incubation time to name but a few, which may be why none of these biomarkers have been brought forward for clinical evaluation [191,203]. Of course, the most significant limitation to this technique is that antibodies do not yet exist for all proteins in the human proteome, which thereby rules ELISA out as a strategy for evaluating many novel protein biomarkers [205]. Protein microarrays can also be used for protein profiling in serum samples. With this technique, thousands of proteins are printed and immobilized onto a glass slide which allows for the simultaneous analysis of serum proteins in a high throughput fashion [206]. This technique is not so extensively used as a means of evaluating PCa biomarkers, although one group did report its application for studying the expression of a HERV-KGAG protein in relation to the clinical progression of PCa. Here it was shown that there was an increased frequency of autoantibodies for HERV-KGAG protein in patients with advanced PCa, making it one of the first reported retroviral cancer antigens in humans [207]. However, as described in the case of ELISAs, reliance on the availability of antibodies and the associated costs are considered to be significant limitations with regards to these microarray-based techniques. As the considerable interest in the development of highly specific and high throughput techniques for biomarker evaluation increases, moving away from traditional antibody-based techniques and branching out into nanotechnology offers a broad spectrum of highly innovative methods to meet the associated requirements for satisfactory biomarker evaluation [206].

#### 6.2.2. Nanotechnology-Based

The development of electrochemical immunosensors has shown great promise for the detection of proteins in clinical applications [208]. Electrochemical immunosensors can provide real time monitoring of biomarkers based on potentiometry and offer simple instrumentation, high sensitivity, fast response time, miniaturization, low cost and point of care applications [209]. To enhance the performance of this immunosensory technology—in which an enzyme is labeled with an antigen—nanomaterials such as gold nanoparticles, carbon nanotubes, silicon nanowires and quantum dots can be used for sensor probes [208]. Quantum dots (QDs) are semiconductor nanocrystals that exhibit unique electrochemiluminescent properties. QDs are applicable for the labeling of peptides, proteins or oligonucleotides and make a desirable alternative to traditionally used organic dyes [210]. This technology has previously been applied for the simultaneous detection of PCa markers in a set of patient serum samples. In this study it was confirmed that the QD-based multiplexed suspension microarray was able to accurately detect either low or high free and total PSA concentrations in clinical serum samples [209]. Carbon nanotubes (CNTs) are made up of thin cylindrical graphite sheets that are optimal for signal amplification due to their fast electron-transfer capabilities and surface area-to-weight ratio. Target analytes are bound to the functionalized CNTs leading to changes in electrical conductance of the device. Proteins or peptides are thereby detected as result of the detectable alteration in electrical conductance caused by the binding of the target analyte [156,210]. CNTs have been successfully utilized in the detection of both PSA and osteopontin (OPN) [211,212]. In the latter study OPN, which is currently under investigation as a potential biomarker for prognosis and diagnosis of PCa, was successfully measured using highly sensitive electrical immunosensors based on single-walled CNTs. With this label-free technique, highly linear and reproducible results were observed for OPN detection over a wide range of OPN concentrations (1 pg/mL–1 mg/mL) in human serum [212]. Another promising PCa biomarker that has also been shown to be selectively detectable through nanotechnology-based technology is matrix metalloproteinase-2 (MMP-2). Using a silicon nanowire-based sensor, MMP-2 was measured in human serum at concentrations ranging from 1 pM to 100 nM [213]. Nanotechnology can also be applied for enrichment of particular proteins or peptides of interest prior to downstream mass spectrometry analysis. As an example, Fredolini et al. demonstrated a novel nanoparticle-based biomarker capture technique to amplify, fractionate and enrich low molecular weight proteins for more sensitive mass spectrometry-based biomarker discovery [188]. Briefly, the core-shell hydrogel nanoparticles described here are capable of complete (in-solution) sequestration of the peptidome while simultaneously performing size sieving enrichments and dramatic concentration of low molecular weight analytes, in one straightforward step. This workflow has already been applied for the identification of PCa-specific candidate biomarkers, revealing a list of novel low molecular weight proteins with potential significance in PCa progression. However, these will warrant further verification and evaluation [188].

#### 6.2.3. Aptamer-Based

Despite the advances in the technologies described above in terms of sensitivity, fast response time, miniaturization, low cost and point of care applications, the availability of antibodies for target proteins still provides a significant limitation. As such, immunosensors based on aptamer interactions are becoming a more favorable approach for sensitive detection of low molecular weight analytes of interest. Aptamers are DNA or RNA molecules with tridimensional conformation that gives them high affinity for specified biomolecules of interest [214]. In contrast to antibodies, aptamers can be easily modified, are smaller in size, cheaper to produce and can be generated against a wide variety of different target molecules [215]. Most aptamers are directly selected against the target analyte and are considered to be more sensitive than an antibody for the same analyte. In fact, problems of capture-reagent cross reactivity and non-specific adsorption to surfaces are greatly reduced with aptamer-based platforms [216]. As such, diagnostic/discovery approaches based on aptamers offer a robust and reliable system for detecting target(s) of interest in direct, indirect and sandwich concepts [214,217]. Aptamer technology has been successfully applied for the detection of PSA in both PCa cells biopsies and human serum. With aptamer-based technology, PSA is detectable at levels as low as fg/mL with high specificity [218]. A modification of this platform is the SOMAscan assay, which uses slow off-rate modified aptamers (SOMAmers). These are single stranded DNA aptamers that contain pyrimidine residues carrying hydrophobic entities at their 5′ position. The affinity of SOMAmers is considerably higher than that of simple RNA or DNA aptamers [219]. Moreover, the platform is highly automated and scalable to allow for high sample throughput [220]. This technology is therefore considered an ideal platform for protein biomarker discovery and evaluation as it has the capacity to detect in excess of 1125 proteins in a single analysis using minimal amounts (<100 μL) of serum [220,221]. In a study by Mehan et al., the SOMAmer platform was used to quantify 1033 proteins simultaneously with sub-pM limits of detection and inter-assay CV of <5% in human serum samples. This analysis resulted in a 7-marker signature for detection of lung cancer in current and former smokers with an AUC of 0.85 for all and 0.93 for squamous cell carcinoma [222]. This study therefore indicates the potential benefits of applying this technology for PCa-related biomarker research.

#### 6.2.4. Mass Spectrometry-Based

For the purposes of verifying the role of identified proteins as potential biomarkers, a targeted proteomic approach provides excellent sensitivity for the detection of potential biomarkers in biological samples [223]. Ultimately, targeted studies are intended to complement discovery-based analysis and facilitate evaluation of protein biomarker expression in biological samples. Selected reaction monitoring (SRM)—otherwise known as multiple reaction monitoring (MRM)—is used for this purpose and enables high throughput, cost-effective assay development for quantification of selected proteins of interest. Essentially, targeted MRM assays can be considered the mass spectrometry equivalent to a western blot or ELISA, only in the case of MRM proteins are identified through the detection of specified combinations of precursor and product ion *m*/*z*’s of preselected proteotypic peptides—thereby eliminating the need for antibodies [224]. MRM infers the ability to quantify hundreds of proteins simultaneously at a low limit of detection with high accuracy. Moreover, MRM-triple quadrupole mass spectrometers also have a wide dynamic range which makes them ideal for analysis of protein expression in serum or plasma digests—the biological fluid of choice for a clinical test [223,225]. With the growing clinical consensus that panels of multiple biomarkers are more likely to achieve adequate clinical specificity and sensitivity for disease diagnosis the development of targeted, high throughput, multiplexed MRM assays is considered to have the greatest potential to bridge the gap between generating panels of biomarker candidates and evaluating their clinical utility in patients [226,227]. Huttenhain et al. recently developed a repository of MRM assays for over 1000 previously identified cancer-associated biomarkers. This study also demonstrated the applicability of MRM assays for reproducible and accurate quantification of biomarker candidates across a large number of patient samples [227]. There are many studies that attest to the effectiveness of combining LC-MS/MS-based discovery with MRM-based multiplexed verification of protein expression in clinical samples [158,228]. The main challenge associated with absolute quantification of proteins of interest using MRM, however, is the requirement for suitable internal standards or heavy labeled peptides which, ideally would have to be synthesized to correspond to each target proteotypic peptide in an MRM-based assay [226]. Although this adds considerably to the cost, the incorporation of stable isotope labeled (SIS) peptides into biological samples does allow for more robust identification and measurement of greater numbers of low abundant protein biomarkers in serum and urine samples [163,229,230].

**Figure 4:**
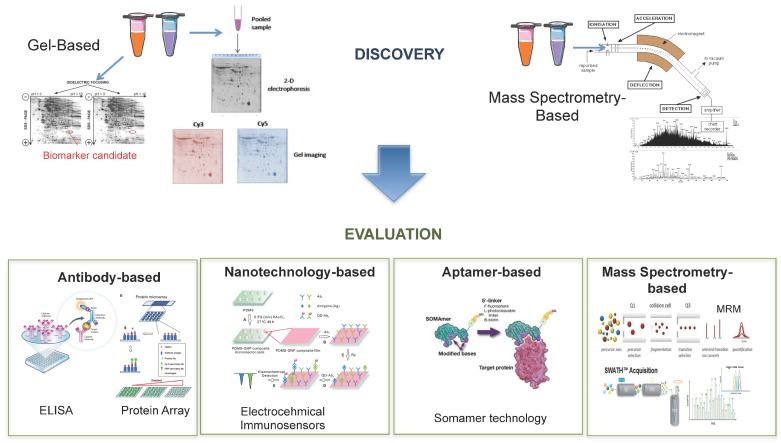
Proteomic Technology for Discovery and Evaluation of Protein Biomarkers.

As detailed throughout this section, proteomic technology has greatly evolved in the last number of years and so accurate measurement of panels of serum and urine proteins is now a feasible and routinely used approach for clinical research. Mass spectrometry has emerged as the forerunner in biomarker discovery and recent advancements have made it possible to identify and quantify the expression of thousands of proteins in biological samples. Validation strategies are moving away from the previous antibody-based gold standard of ELISA with the emergence of nano-technologies and aptamer-based technologies making it possible to measure low abundant proteins in serum and urine samples (Figure 4). However, with regards to PCa and, indeed, most other cancers, targeted mass spectrometry methods have again been at the forefront of biomarker verification and validation studies.

## 7. Clinical Evaluation of Prostate Cancer Biomarkers

### 7.1. Evaluation of Emerging Prostate Cancer Biomarkers

**Figure 5:**
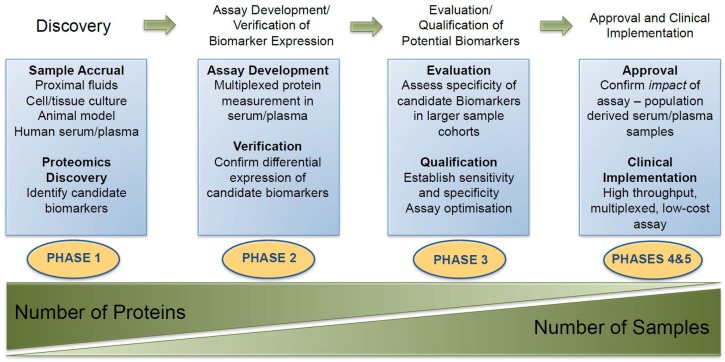
Discovery and Clinical Implementation of Protein Biomarkers: outline of the process involved in bringing candidate protein biomarkers through from the discovery phase, evaluation and ultimately to adoption as a clinical assay for subsequent validation (Figure adapted from Rifai et al., 2006 [116].

The use and implementation of high throughput, sensitive and robust proteomic technologies, as described throughout this review, for biomarker research enable a more personalized approach for disease detection and treatment [112]. Indeed, the advances made in proteomics technology over the last number years has led to the identification of over 200 protein biomarkers to date [112,231,232,233,234,235,236,237]. However, while some of these may have been evaluated in biological samples from additional patient cohorts, none have yet been formally validated for use as a clinical assay. This is not overly surprising when one considers that the FDA has only clinically approved little over 24 biomarkers for any cancer, and almost none of them are actually used in standard clinical practice [238]. Even PSA, which is the most widely used blood-based biomarker for any disease, took more than ten years to transition from a potential biomarker candidate to a clinically-used test for PCa detection [239]. Some would argue that this is due, in part, to the fact that scientists working in the field of biomarker discovery actually have limited knowledge of the analytical, diagnostic and regulatory requirements required for a biomarker to qualify for use as part of a clinical assay [240]. Clinical evaluation of a disease biomarker is a time-consuming, arduous and expensive process. In 2001, following establishment of the ‘four phase’ guideline for clinical trial, the NCI EDRN identified five phases of biomarker development for the early detection of cancer (Figure 5). Phase one represents the initial discovery phase, where molecular discrepancies between tumor and non-tumor lead to the identification of a potential biomarker. In phase two, biomarker expression is evaluated in relatively small or moderately sized cohorts. Phase three is defined as the ‘retrospective longitudinal evaluation’ of biomarkers, while phases four and five involve prospective evaluation of potential biomarkers as a screening test. The impact of a biomarker/panel of biomarkers in the general population, in terms of both mortality and cost, is also assessed in these latter stages. As phases four and five involve healthy individuals at the beginning, they also take much more time to complete and require a large number of participants [239,241]. Despite the widespread efforts being made worldwide to identify clinically useful biomarkers for PCa, none have made it past the studies required for phase 5. So, although proteomic technology has hugely advanced our abilities to generate vast amounts of biologically relevant data from biological samples, emerging biomarkers repeatedly fail to overcome some of the many bottlenecks associated with clinical evaluation.

### 7.2. Overcoming Bottlenecks Associated with Clinical Evaluation of Prostate Cancer Biomarkers

A considerable investment of time and money will be required for a biomarker to make it through all five phases of biomarker discovery and evaluation described by Pepe et al. [241]. Therefore, careful planning at every stage of the process is essential. With regards to protein biomarkers, standardization of pre-analytical steps is crucial for robustness and reproducibility of the final assay. This has been highlighted in an investigation reported by Addona et al., in which a multi-laboratory study was conducted to assess reproducibility, recovery, linear dynamic range and limits of detection and quantification of multiplexed MRM-based assays conducted by NCI-CPTAC [242]. Aside from assay robustness, another major pitfall that must be overcome is that of false discovery. One of the main reasons that potential biomarkers fail to make it into use as a clinical assay is that many of them are deemed significant in an initial discovery cohort but are subsequently found to not be significant in the proceeding evaluation studies. Appropriate statistical analysis of proteomic data—especially the large amount that is generated by mass spectrometry-based and aptamer-based technology—is an area where many biologists fall short. For example, Hernandez et al. have shown that the commonly used approach of pre-filtering initial discovery data using ANOVA and correction methods such as Bonferroni and FDR rarely improves the accuracy of biomarker selection [243]. Indeed, it is suggested here that only when the data is not pre-filtered can the quality of a biomarker(s) be accurately judged for predictive capacity. ANOVA filtering should in fact be performed on a separate cohort to that which was used to assess the performance of the chosen biomarker or panel of biomarkers [243]. This author also suggests that the initial proteomic discovery experiments be performed on sufficiently powered (at least 50) sample numbers to minimize the effects of over-fitting and improve the quality of performance metrics [243]. It is fair to say that availability of sufficient sample numbers at this stage and, more importantly, at the subsequent evaluation stages is a major limitation in biomarker research. However, investing the time and money in a statistically powered study at this early stage will ultimately avoid any wasted expense in continuing to evaluate a non-predictive biomarker and/or biomarker panel.

**Figure 6:**
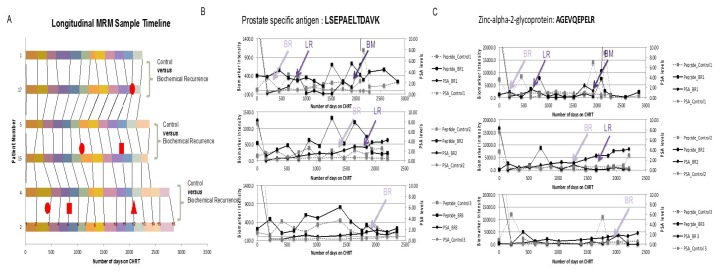
Longitudinal Evaluation of Candidate Prostate Cancer Biomarkers in Patient Serum Samples: Serum samples collected from patients who had failed treatment with CHRT (*n* = 3) and time-matched controls still responsive to CHRT (*n* = 3) (**A**) were used to longitudinally evaluate the expression of multiple markers of PCa, including PSA (**B**) and zinc-slpha-2-glycoprotein (**C**) via MRM measurement of proteotypic peptides. Figure adapted from Tonry et al. 2015 [247].

The third phase described by Pepe et al. for the clinical evaluation of protein biomarkers involves retrospective longitudinal evaluation of potential biomarkers [241]. A significant limitation at this stage of the process is access to high-quality longitudinal samples from a patient cohort. The importance of longitudinal evaluation, however, cannot be ignored, especially in PCa, which is a highly heterogeneous disease with a prolonged time course [244]. The value of longitudinal evaluation of patient cohorts has been highlighted in a number of studies related to PCa [201,245]. For example, in 2009, Christensen et al. investigated longitudinal cytokine expression in PCa patients undergoing intensity-modulated radiotherapy (IMRT) over the course of a year. Although this study alluded to a relationship between IMRT toxicity and cytokine expression, future studies would be warranted to determine the time course for serum cytokine changes after radiation exposure in a larger cohort [246]. A more recent study availed of a unique cohort of patients who are participating in a non-interventional clinical trial and being treated with CHRT. Samples from patients who had failed treatment with CHRT over the course of the trial (approximately 7 years) and time-matched controls (non-failures) from the same cohort were used to identify potential biomarkers of treatment failure at both baseline and time of failure. Identified biomarkers, as well as PSA, were then evaluated longitudinally in the failure and control patients in samples that had been collected throughout their participation in the trial (Figure 6) [247]. This study highlighted the need to consider the inherent inter-patient variability associated with PCa when seeking to evaluate the utility of potential PCa biomarkers. Access to high quality longitudinal patient samples, collected under clinical trial governance, can offer much more accurate insight into the potential predictive and/or prognostic capacity of biomarkers of interest, in individual patients. As such, in order to achieve meaningful translational advances in PCa biomarker development, researchers much shift their focus towards well-designed, collaborative efforts to ensure that verification and subsequent validation studies are suitably powered and are not at risk for false discoveries [248].

### 7.3. Potential for Routine Use of MS Technologies for Clinical Diagnostics in PCa

As outlined previously in this review, the standard approach for biomarker development involves the identification of a crude list of biomarker candidates obtained from either a representative set of clinical samples or model cell system using comparative profiling techniques such as 2D DIGE or LC-MS/MS. The next step is to verify the crude list of biomarkers in individual clinical samples of blood or tissue. Traditionally, this would have been done by antibody-based techniques such as Western blot or ELISA but, as outlined in previous sections, MS-based approaches such as MRM are now considered a much more favorable option for this process. Among its benefits, MRM is a high throughput technique that can measure up to 50 proteins with an injection of 1–2 μg peptide from less than 20 μL blood [249]. Indeed, with the advent of scheduled MRM, over 100 target proteins can now be targeted in a single assay [250]. There are of course a number of obstacles with regards to the routine use of MRM for clinical validation of biomarkers and these are in regard to (i) high throughout and reproducible sample preparation; (ii) selection of appropriate transitions for each peptide (protein) target and (iii) the cost associated with the development of assays using stable isotope peptides for internal standardization and quantification [251]. The first of these obstacles can be addressed by implementation of robust standard operating procedures and automation of sample preparation techniques. Indeed, a number of groups have investigated the robustness and reproducibility of the entire MRM analysis workflow across multiple sites [250,252]. With regards to the selection of appropriate transitions, there are a number of publicly available tools available that can be used for the design of MRM assays. These include PASSEL, NIST peptide library and SRM atlas. Finally, the development of mass differential tags for relative and absolute quantitation, tandem mass tag and 18O labeling and dimethyl labeling circumvent the need to purchase stable isotope internal standards, thus allowing for effective and inexpensive normalization and quantitation of MRM data [251]. Moreover, workflow innovations such as SISCAPA which detect low abundance proteins in blood [253] and software such as Skyline and MRM3 [254,255] render the development and use of MRM assays easier to use for less trained individuals [256].

The future of mass spectrometry in the clinical setting has been a topical debate among expert researchers in the field of clinical research in recent years. There is a shared opinion that, since mass spectrometry technology is evolving rapidly with new innovations in workflow, software, hardware and reagents, automated immunoassays will be replaced with MS assays in clinical laboratories [248]. Moreover, it is believed that this technology will provide added benefit to genomic tests that cannot provide information on clinically important protein isoforms [248]. These opinions are supported by a number of commercially available MS-based diagnostic tests. One of the most established of these is the LC-MS/MS measurement of 25-hydroxy metabolites of Vitamin D2 and Vitamin D3. Unlike the previous automated immunoassay platforms that measured Vitamin D levels as a whole, this MS-based assay ensures that both endogenous and exogenous Vitamin D metabolites are measured equimolarly. This assay is now in routine diagnostic use and the automated LC-MS/MS system allows up to 180 tests to be performed in a 24 h period [257,258]. A test has also been developed to measure carbohydrate deficient transferrin, a biochemical marker for congenital disorders of glycosylation. For this protein, the previous affinity chromatography IEF method has been replaced by the development of an automated LC-MS/MS method capable of analyzing over 100 samples in one day [259]. Mass spectrometry coupled to immunoaffinity separations can provide an efficient means for simultaneous detection and quantification of protein variants and this has been applied to establish an MS-based clinical assay for measurement of variants of cystatin C—a marker of renal failure among other pathological conditions [260]. A similar assay has also been established for measurement of beta-2-glycoprotein in plasma samples. As well as being an FDA approved biomarker for active rheumatoid arthritis and kidney disease, this protein has also been heavily associated with PCa progression [261]. A number of MS-based assays are also now offered for the detection of insulin resistance and type-2 diabetes by measurement of retinol binding protein [262], insulin-like growth factor I and II [263] and insulin [264,265]. An isotope dilution LC-MS/MS method has also been developed for the detection of angiotensin in blood—an important protein marker of hypertension [266]. Two commercially available MS-based have been developed for improved management of lung cancer—Veristat and Express Lung. The Veristat assay is a MALDI-MS algorithm based on 8 distinct *m*/*z* features and has been validated as a clinically useful serum protein test [267,268,269]. The Express Lung tests is an MRM-based assay measuring five diagnostic and six normalisation proteins and has also been validated as a proteomic classifier for identification of benign lung nodules with a high negative predictive value [270]. Nuclea Biotechnologies also offer LC-MS/MS-based tests to measure serum levels of c-peptide, proinsulin, apoplipoprotein A1 and Apolipoprotein B. Although the tests described here have not yet been FDA approved, they are currently categorized as lab-developed tests LDTs and have been developed and characterized under CLIA requirements. In addition to the reported success of these assays, the increasingly significant role of mass spectrometry in the clinical diagnostics setting is reflected in the development of consortia—notably the NCI CPTAC and the EDRN—which have placed a heavy emphasis on good experimental design and the need to reduce false discovery (as discussed in Section 7.2) [195]. Overall, the recent advancements in MS technology and the successful implementation of CLIA approved MS assays for diagnosis of various other disease conditions, would indicate that routine MS-based measurements of PCa biomarkers could indeed be translated to a clinical setting.

## 8. Conclusions

There is without doubt an on-going need to identify biomarkers for PCa that could be measured as part of a non-invasive clinical assay and used to improve disease management and treatment of PCa patients. The limitations of PSA and most other protein biomarkers identified to date have led to the consensus that multiplexed measurements of multiple biomarkers—as part of a panel—in a single assay would be of the greatest utility [271]. The lack of a technologies capable of verifying or evaluating the large number of potential biomarkers that are identified in discovery experiments was once considered the major bottleneck in the biomarker development pipeline [271]. However, as highlighted in this review, the field of biomarker research is no longer limited by lack of methods for large-scale, high-throughput, robust and reproducible biomarker identification and evaluation. Despite these advances, the major investments made in these technologies (in academia and industry) have been rewarded with limited return in terms of delivery of effective clinical tests. The failure of most individual new biomarkers to make it to the clinic can, to some extent, be attributed to the fact that many fail to offer a greater clinical value and impact than existing tests, despite the latter’s known limitations. The expectation that this situation will be remedied by the development of multiplexed tests has to some extent driven the development of high-throughput, multiplexed proteomics technologies suitable for the discovery and evaluation phases of biomarker assay development. One such mass spectrometry-based technique—MRM—offers great potential due to its ability to provide robust, sensitive, quantitative, specific and high-throughput measurement of panels of protein biomarkers in complex biospecimens. Furthermore, MRM is already routinely used in a clinical setting and various CLIA-approved MRM-based assays are now available as diagnostic tests [272]. In contrast to immune-based technologies, mass spectrometry based tests also offer a potential greater return on investment. This was highlighted by Anderson et al., who concluded that the cost of generating and applying high quality MS-based (MRM) assays for approximately 50–100 good biomarker candidates could be estimated at about $1.5 million (15–30 k/protein), in contrast to the $2.2 million required to develop ELISA assays for six candidate biomarkers (>353 k/protein). These authors estimated the cost of using an MS strategy to bring a selection of candidate biomarkers through the whole biomarker development pipeline to be $4 million and suggest that it would take about 4 years [273].

We suggest that these technological advancements for multiplexed assays demand that more consideration be given to very careful study design in all aspects of assay development to ensure that they are sensitive, specific and precise while maintaining the robustness and reproducibility required for clinical use, potentially across multiple sites. Candidate biomarker verification has become a critical step in the biomarker development pipeline. It is evident that, ideally, only analytically verified candidates should be brought forward for further development and the stringency of this selection process should be aligned with the cost and effort required for clinical evaluation of the candidate biomarkers. This approach is likely to reduce the often wasted time and cost in attempting to evaluate biomarkers that are unlikely to be used as part of clinical assay [274]. Ultimately, access to large sample numbers (including longitudinal samples), rigorous statistical analysis to avoid over-fitting of data and an overall strategy focused on providing assays that fulfill compelling clinical needs will be required if protein biomarker development is going to have an impact on improving the diagnosis and treatment of PCa patients. Researchers must also give careful consideration as to where the true clinical need for protein biomarkers lies for PCa. Highly sensitive detection of PCa is already achievable with PSA and we have learned, after 30 years of usage, that such sensitivity has resulted in the over-treatment of men with PCa—often with adverse effects on their quality of life. We propose that a test that is much needed for PCa is one that would reduce the number of men diagnosed with the disease that are treated unnecessarily. Therefore, biomarker research in PCa should be aimed more towards stratifying patients with PCa for more appropriate and personalized management of the disease.

## Figures and Tables

**Table 1:** European Association of Urology Guidelines for Prostate Cancer Screening, Diagnosis and Treatment.

Category	Screening and Diagnosis
Epidemiology	214 cases per 1000 men
Risk Factors	Increasing age, ethnic origin and heredity
Classifications	Union Internationale Contre le Cancer 2010 TNM
	Gleason scoring recommended for grading
Prostate Cancer Screening	1. Routine screening not recommended for men ages 40–54 years
	2. Recommended shared decision making for men aged 55–69 years
	3. Routine screening interval of ≥2 years in men who decide on screening
	4. Routine screening not recommended for men ≥70 years or with life expectancy <10–15 years
Diagnosis and Staging	1. Abnormal DRE/elevated PSA (cut-off level for normal PSA not yet determined)
	2. Diagnosis depends on histopathologic confirmation
	3. TRUS-guided systemic biopsy with ≥10 systemic, laterally directed cores
	4. One set of repeat biopsies recommended in cases with persistent indication for prostate biopsy (abnormal DRE, elevated PSA, ASAP, multifocal PIN)
	5. MRI to investigate anteriorly located PCa if biopsy negative and clinical indications of PCa persist
	**Primary Local Treatment**
Active Surveillance	1. >10 years life expectancy
	2. Stage T1–T2
	3. PSA ≤ 10 ng/mL
	4. Biopsy Gleason score <6
	5. ≤2 positive biopsies
	6. ≤50% cancer per biopsy
Radical Prostatectomy	1. Patients with life expectancy >10 years
	2. In patients with high-risk localised PCa, life expectancy >10 years, offered in multimodality setting
	3. In patients with high risk locally advanced with life expectancy >10, may be offered in multimodality setting
Radiation therapy (low risk)	Dose of 74–78 Gy
Radiation Therapy (intermediate risk)	EBRT dose of 76–78 Gy in combination with short-term (4–6 months) ADT
Radiation therapy (high risk, localised)	EBRT dose 76–78 Gy in combination with long-term (2–3 years) ADT
Transperineal brachytherapy as monotherapy	1. Stage cT1c-T2a, NOMO ^1^
	2. Gleason score ≤7 on at least 12 random biopsies
	3. Initial PSA ≤10 ng/mL
	3. ≤50% biopsy cores involved with cancer
	4. A prostate volume of <50 mL
	5. A good International Prostate Symptom Score (≤17)
	6. No previous transurethral resection of the prostate

DRE = digital rectal exam; PSA = prostate specific antigen; TRUS = transrectal ultrasound; ASAP = atypical small acinar proliferation in the prostate; PIN = prostatic intraepithelial neoplasia; MRI = molecular resonance imaging; ADT = androgen deprivation therapy; EBRT: external beam radiation therapy; ^1^ PCa tumor staging described in supplementary data Table S1.

**Table 2:** Newly Emerging Tests for Prostate Cancer ^1^.

Assay	Marker Description	Assay Type	Biomaterial	FDA Approved
**Tissue-Based**				
**Oncotype DX**	17 genes	RT-PCR	Fixed paraffin embedded needle core biopsy	No
**Prolaris**	46 genes	RNA expression	Formalin-fixed paraffin embedded needle core biopsy	No
**ProMark**	8 proteins	Immunofluorescent imaging	Formalin-fixed paraffin embedded needle core biopsy	No
**Decipher**	22 coding and non-coding RNAs	Whole-transcriptome microarray	Formalin-fixed paraffin embedded needle core biopsy	No
**Confirm MDx**	3 genes	Quantitative methylation-specific PCR	Prostate needle core biopsy	No
**PCMT**	mtDNA deletions	Quantitative PCR (specific for mtDNA)	Prostate needle core biopsy	No
**Fluid-Based**				
**phi**	PSA, fPSA, p2PSA	Multi-analyte Immunoassay	Serum	No
**4K score**	total PSA, fPSA, intact PSA, hK2	Multi-analyte Immunoassay	Plasma	No
**Progensa (PCA3)**	PSA and PCA3 mRNA	in vitro RNA TMA assay	Post-DRE first void urine	Only when repeat biopsy considered
**SelectMDx**	HOXC6, DLX1, KLK3	Reverse Transcription PCR (RT-PCR)	Post-DRE first void urine	No
**MiPS**	PSA,PCA3 and TMPRSS2:ERG mRNAs	in vitro RNA TMA and Hybrid Protection Assay (HPA)	Post-DRE first void urine	No
**Prostarix**	4 amino acids: sarcosine, alanine, glycine and glutamate	Liquid chromatography and mass spectrometry	Post-DRE urine	No
**ExoDx Prostate (IntelliScore)**	Exosomal RNA (ERG, PCA3, SPDEF)	RT-PCR	Urine	No

^1^ Table adapted from Falzarano et al. [94].

**Table 3:** Selected Publications Related to Prostate Cancer and Proteomics research over the last ten years.

Reference	Title	Marker(s)
Webber, JP et al. *Oncotarget* 2016 [117]	Prostate stromal cell proteomics analysis discriminates normal from tumour reactive stromal phenotypes	Proteins including TAGLN, VDAC1, VDAC2, ALDH1A1
Adeola, HA et al. *Oncotarget* 2016 [118]	Novel potential serological prostate cancer biomarkers using CT100+ cancer antigen microarray platform in a multi-cultural South African cohort.	41 antigen biomarkers including GAGE1, ROPN1, SPANXA1, PRKCZ, MAGEB1, p53, S15A, S46A, FGFR2, COL6A1, CALM1
Li, Q et al. *Int. J. Oncol.* 2016 [119]	Quantitative proteomic study of human prostate cancer cells with different metastatic potentials	SETDB1
Ino, Y et al. *Proteomics* 2016 [120]	Phosphoproteome analysis demonstrates the potential role of THRAP3 phosphorylation in androgen-independent prostate cancer cell growth.	THRAP3
Kazuno, S et al. *Cancer Med.* 2016 [121]	Glycosylation status of serum immunoglobulin G in patients with prostate diseases	Glycosylation changes in IgG
Stone, L. *Nat. Rev. Urol.* 2016 [122]	Prostate cancer: Proteomics provides a prognostic marker.	-
Davalieva, K et al. *Prostate* 2015 [123]	Proteomics analysis of malignant and benign prostate tissue by 2D DIGE/MS reveals new insights into proteins involved in prostate cancer	9 proteins (CSNK1A1, ARID5B, LYPLA1, PSMB6, RABEP1, TALDO1, UBE2N, PPP1CB, and SERPINB1)
Arner, P et al. *PLoS ONE* 2015 [124]	Circulating carnosine dipeptidase 1 associates with weight loss and poor prognosis in gastrointestinal cancer	CNDP1
Shipitsin, M et al. *Br. J. Cancer* 2014 [90]	Identification of proteomic biomarkers predicting prostate cancer aggressiveness and lethality despite biopsy-sampling error	12 proteins (ACTN1, CUL2, DERL1, FUS, HSPA9, PDSS2, PLAG1, pS6, SMAD2, SMAD4, VDAC1, YBX1)
Bergamini, S et al. *Proteome Sci.* 2014 [125]	Inflammation: an important parameter in the search of prostate cancer biomarkers.	9 Proteins (F2, C4B, C3, AZGP1, HPX, SERPINC1, SERPINF1, HP, SAA1)
Pallua, JD et al. *J. Proteomics* 2013 [126]	MALDI-MS tissue imaging identification of biliverdin reductase B overexpression in prostate cancer	BLVRB
Leymarie, N et al. *Mol. Cell Proteom.* 2013 [127]	Interlaboratory study on differential analysis of protein glycosylation by mass spectrometry: the ABRF glycoprotein research multi-institutional study 2012	Glycoforms of PSA
Jiang, FN et al. *PLoS ONE* 2013 [128]	An integrative proteomics and interaction network-based classifier for prostate cancer diagnosis	3 proteins (PTEN, SFPQ, HDAC1)
Han, ZD et al. *Med. Oncol.* 2012 [129]	Identification of novel serological tumor markers for human prostate cancer using integrative transcriptome and proteome analysis	IMPDH2
Endoh, K et al. *Prostate* 2012 [130]	Identification of phosphorylated proteins involved in the oncogenesis of prostate cancer via Pin1-proteomic analysis	TFG
Cheng, HL et al. *Proteom. Clin. Appl.* 2011 [131]	Urinary CD14 as a potential biomarker for benign prostatic hyperplasia—discovery by combining MALDI-TOF-based biostatistics and ESI-MS/MS-based stable-isotope labeling	CD14
Alaiya, AA et al. *Int. J. Oncol.* 2011 [132]	Proteomics-based signature for human benign prostate hyperplasia and prostate adenocarcinoma	15 proteins (TPM1, PHB, KRT8, TUBB2, DES, Glycerol 3 phosphate, P4HB, EHHADH, HSPA5, KRT18, SERPINA1, CKB, HSPA8, ATP5B, ANXA4
True, LD et al. *Mod. Pathol.* 2010 [133]	CD90/THY1 is overexpressed in prostate cancer-associated fibroblasts and could serve as a cancer biomarker	CD90/THY1
Valmu, L et al. *Methods Mol. Biol.* 2010 [134]	Proteomic analysis of pancreatic secretory trypsin inhibitor/tumor-associated trypsin inhibitor from urine of patients with pancreatitis or prostate cancer	PSTI
Thoenes, L et al. *J. Proteom.* 2010 [135]	In vivo chemoresistance of prostate cancer in metronomic cyclophosphamide therapy	3 proteins (TXN, CTSB, ANXA3)
Van der Deen, M et al. *J. Cell Biochem.* 2010 [136]	The cancer-related Runx2 protein enhances cell growth and responses to androgen and TGF-beta in prostate cancer cells	Runx2
Sardana, G et al. J *Proteome Res.* 2008 [137]	Proteomic analysis of conditioned media from the PC3, LNCaP, and 22Rv1 prostate cancer cell lines: discovery and validation of candidate prostate cancer biomarkers	4 proteins (FST, CXCL16, PTX3, SPON2)
Saito, S et al. *Int. J. Cancer* 2008 [138]	Haptoglobin-beta chain defined by monoclonal antibody RM2 as a novel serum marker for prostate cancer	RM2
Ummanni, R et al. *Cancer Lett.* 2008 [139]	Prohibitin identified by proteomic analysis of prostate biopsies distinguishes hyperplasia and cancer	PHB
Huang, D et al. *Cancer Epidemiol. Biomark. Prev.* 2007 [140]	Quantitative fluorescence imaging analysis for cancer biomarker discovery: application to beta-catenin in archived prostate specimens	CTNNB1
Ruan, W et al. *Mol. Cell Proteom.* 2006 [141]	Identification of clinically significant tumor antigens by selecting phage antibody library on tumor cells in situ using laser capture microdissection	ALCAM, MEMD, CD166
Johansson, B et al. *Prostate* 2006 [142]	Proteomic comparison of prostate cancer cell lines LNCaP-FGC and LNCaP-r reveals heatshock protein 60 as a marker for prostate malignancy	HSP60
Lam YW et al. *Proteomics* 2005 [143]	Mass profiling-directed isolation and identification of a stage-specific serologic protein biomarker of advanced prostate cancer	PF4

**Table 4:** Considerations for Sample Selection for Biomarker Discovery ^1^.

	Tissue		Body Fluids		
	Biopsy	Needle Biopsy	Serum & Plasma	Urine	Prostatic Fluid and Seminal Plasma
**Advantages**	Direct analysis of tumor protein expression/activation		Non-invasive collection	Non-invasive collection	Minimally invasive collection
	Diagnostic markers		Fast and low-cost sample preparation	High volume	Rich in prostate-derived proteins
	Prognostic markers		Diagnostic markers	Rich in prostate-derived proteins	Fast and low-cost sample preparation
	Most useful for patient stratification in terms of response to therapy		Prognostic markers	Fast and low-cost sample preparation	Diagnostic markers
	-		-	Diagnostic markers	Prognostic markers
	-		-	Prognostic markers	
**Limitations**	Invasive collection		Low abundance of potential biomarkers	Low abundance of potential biomarkers	Low abundance of potential biomarkers
	Limited quantity		Dynamic concentration range	Dynamic concentration range	Dynamic concentration range
	Must be snap-frozen within 30 minutes from collection		Intra and inter-patient variability in composition	Intra and inter-patient variability in composition	Intra and inter-patient variability in composition
	Complicated sample preparation		-	Variability in sample collection	-

^1^ Table adapted from Pin et al., 2013 [95].

## Supplementary Materials

Supplementary File 1

## Abbreviations

The following abbreviations are used in this manuscript:

BPH	Benign Prostatic Hyperplasia
PCa	Prostate Cancer
AR	Androgen Receptor
PSA	Prostate Specific Antigen
US	United States
CE-IVD	European Conformity—In Vitro Diagnostics
CHRT	Combined hormone and radiation therapy
ADT	Androgen deprivation therapy
AS	Active surveillance
PRIAS	Prostate cancer research international active surveillance
SCAN	Scotland cancer research
CCO	Cancer care Ontario
GAP	Global action plan
fPSA	free PSA
hK2	Hexokinase 2
MSMB	microseminoprotein beta
MIC1	Macrophage inhibitor cytokine 1
DRE	Digital rectal exam
PSAV	PSA velocity
PSADT	PSA doubling time
p2PSA	[-2] proenzyme PSA
PHI	Prostate health index
PTEN	Phosphate and tensin homologue
FFPE	Fresh frozen paraffin embedded
RT-PCR	Real time polymerase chain reaction
CCP	Cell cycle progression
TURP	Transurethral resection of the prostate
PCA3	Prostate cancer antigen 3
CLIA	Clinical laboratory improvement amendments
LCM	Laser capture microdissection
RBC	Red blood cells
WBC	White blood cells
EPS	Expressed prostatic secretion
2D-PAGE	Two dimensional poly-acrylamide gel electrophoresis
DIGE	Differential gel electrophoresis
SDS	Sodium dodecyl sulfate
MS	Mass spectrometry
LC-MS/MS	Liquid chromatography tandem mass spectrometry
SWATH	Sequential window acquisition of all theoretical ions
MRM	Multiple reaction monitoring
ELISA	Enzyme-linked
QD	Quantum dot
CNT	Carbon nanotube
SRM	Selected reaction monitoring
NCI EDRN	National Cancer Institute—The early detection research network
NCI CPTAC	National Cancer Institute—Cancer clinical proteomics research
ANOVA	Analysis of variance
FDR	False discovery rate
IMRT	Intensity modulated radiation therapy
IPSS	International prostate symptom score
SISCAPA	Stable isotope standard capture with anti-peptide antibodies
MALDI-MS	Matrix assisted laser desorption/ionization mass spectrometry
