# Supplementary material for: The Role of Proteomics in Biomarker Development for Improved Patient Diagnosis and Clinical Decision Making in Prostate Cancer
Source: Diagnostics (Basel). 2016 Jul 18;6(3):27. doi: 10.3390/diagnostics6030027 (PMC5039561; doi:10.3390/diagnostics6030027)
Supplement: Supplementary File 1 [file diagnostics-06-00027-s001.pdf]

Supplementary Materials: The Role of Proteomics in Biomarker Development for Improved Patient Diagnosis and Clinical Decision Making in Prostate Cancer

Claire L. Tonry, Emma Leacy, Cinzia Raso, Stephen P. Finn, John Armstrong and Stephen R. Pennington

Table S1. Tumor Staging in Prostate Cancer [1] [1].

	**Primary Tumor (T)**
**TX**	Primary tumor cannot be assessed
**T0**	No evidence of primary tumor
**T1**	Clinically inapparent tumor not palpable or visible by imaging
**T1a**	Tumor incidental histologic finding in ≤5% of tissue resected
**T1b**	Tumor incidental histologic finding in >5% of tissue resected
**T1c**	Tumor identified by needle biopsy (because of elevated prostate specific antigen [PSA] level)
**T2**	Tumor confined within prostate; tumors found in 1 or both lobes by needle biopsy but not palpable or reliably visible by imaging
**T2a**	Tumor involves one-half of 1 lobe or less
**T2b**	Tumor involves more than one-half of 1 lobe but not both lobes
**T2c**	Tumor involves both lobes
**T3**	Tumor extends through the prostatic capsule; invasion into the prostatic apex, or the prostatic capsule is classified not as T3 but as T2
**T3a**	Extracapsular extension (unilateral or bilateral)
**T3b**	Tumor invading seminal vesicle(s)
**T4**	Tumor fixed or invades adjacent structures other than seminal vesicles (e.g., bladder, levator muscles, and/or pelvic wall)
	**Pathologic (pT) ***
**pT2**	Organ confined
**pT2a**	Unilateral, involving one-half of 1 lobe or less
**pT2b**	Unilateral, involving more than one-half of 1 lobe but not both lobes
**pT2c**	Bilateral disease
**pT3**	Extraprostatic extension
**pT3a**	Extraprostatic extension or microscopic invasion of the bladder neck
**pT3b**	Seminal vesicle invasion
**pT4**	Invasion of the bladder and rectum
*** Positive surgical margin should be indicated by an R1 descriptor (residual microscopic disease)**	
	**Regional lymph nodes (N)**
Clinical	
**NX**	Regional lymph nodes were not assessed
**N0**	No regional lymph node metastasis
**N1**	Metastasis in regional lymph node(s)
Pathologic	
**PNX**	Regional nodes not sampled
**pN0**	No positive regional nodes
**pN1**	Metastases in regional nodes(s)
	**Distant metastasis (M) ***
**M0**	No distant metastasis

**M1**	Distant metastasis
**M1a**	Nonregional lymph nodes(s)
**M1b**	Bone(s)
**M1c**	Other site(s) with or without bone disease

[1] Table adapted from National Comprehensive Cancer Network.

Reference

1. NCCN Clinical Practice Guidelines in Oncology: Prostate Cancer. V.1.2015. Available online: http://www.nccn.org/professionals/physician_gls/pdf/prostate.pdf (accessed on 30 December 2015).
